# (2,2′-Bipyrid­yl)bis­[*N*,*N*-bis­(2-hydroxy­ethyl)dithio­carbamato-κ^2^ *S*,*S*′]cadmium(II)

**DOI:** 10.1107/S1600536809049678

**Published:** 2009-11-25

**Authors:** Juyoung C. Song, Edward R. T. Tiekink

**Affiliations:** a Department of Chemistry, The University of Texas at San Antonio, One UTSA Circle, San Antonio, Texas 78249-0698, USA; b Department of Chemistry, University of Malaya, 50603 Kuala Lumpur, Malaysia

## Abstract

The title compound, [Cd(C_5_H_10_NO_2_S_2_)_2_(C_10_H_8_N_2_)], features a trigonal-prismatic coordination geometry for the Cd^II^ ion, based on an N_2_S_4_ donor set defined by two chelating dithio­carbamate ligands and a 2,2′-bipyridyl ligand. In the crystal, extensive O—H⋯O hydrogen bonding results in the formation of 12-membered {⋯HO}_6_ synthons and one-dimensional supra­molecular chains with further O—H⋯S inter­actions providing additional stability to the linear chain with base vector [01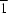].

## Related literature

For background to supra­molecular polymers of zinc-triad 1,1-dithiol­ates, see: Tiekink (2003 ▶); Lai *et al.* (2002 ▶); Chen *et al.* (2006 ▶); Benson *et al.* (2007 ▶). For the synthesis, see: Lai & Tiekink (2004 ▶). *Note added in proof*: a room temperature determination of the same structure has been reported by [Deng, Y.-H., Liu, J., Li, N., Yang, Y.-L. & Ma, H.-W. (2007). *Acta Chim. Sin.* **65**, 2868–2874].
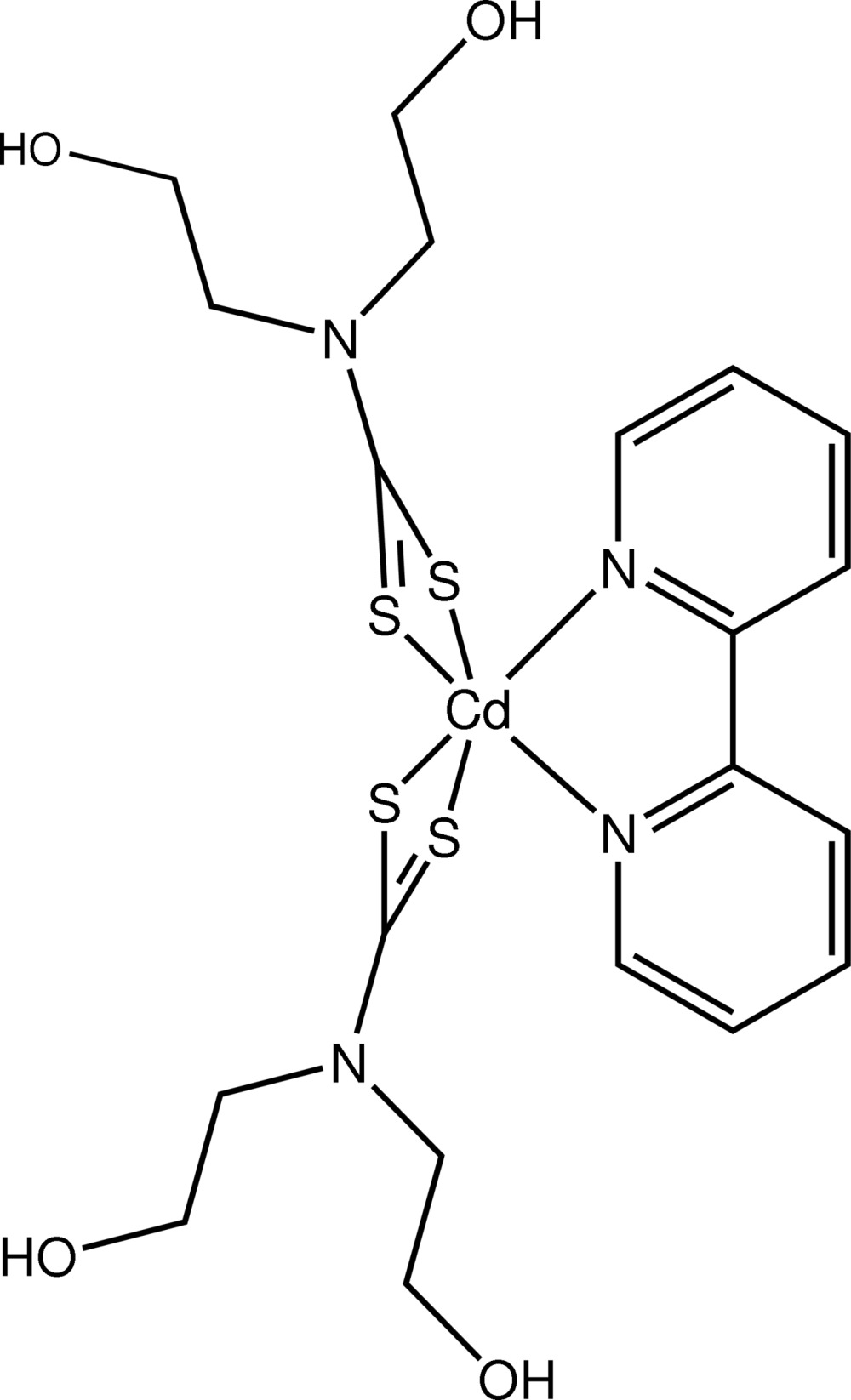


## Experimental

### 

#### Crystal data

[Cd(C_5_H_10_NO_2_S_2_)_2_(C_10_H_8_N_2_)]
*M* *_r_* = 629.10
Triclinic, 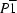

*a* = 10.077 (2) Å
*b* = 11.568 (2) Å
*c* = 11.676 (2) Å
α = 70.85 (3)°
β = 85.86 (3)°
γ = 81.21 (3)°
*V* = 1270.3 (4) Å^3^
*Z* = 2
Mo *K*α radiation
μ = 1.22 mm^−1^
*T* = 173 K
0.33 × 0.21 × 0.03 mm

#### Data collection

Rigaku AFC12K/SATURN724 diffractometer
Absorption correction: multi-scan (*ABSCOR*; Higashi, 1995 ▶) *T*_min_ = 0.836, *T*_max_ = 1
25287 measured reflections
5262 independent reflections
4944 reflections with *I* > 2σ(*I*)
*R*_int_ = 0.073

#### Refinement

*R*[*F* ^2^ > 2σ(*F* ^2^)] = 0.053
*wR*(*F* ^2^) = 0.116
*S* = 1.13
5262 reflections
310 parameters
4 restraints
H-atom parameters constrained
Δρ_max_ = 0.56 e Å^−3^
Δρ_min_ = −0.98 e Å^−3^

### 

Data collection: *CrystalClear* (Rigaku/MSC, 2005 ▶); cell refinement: *CrystalClear*; data reduction: *CrystalClear*; program(s) used to solve structure: *PATTY* in *DIRDIF92* (Beurskens *et al.*, 1992 ▶); program(s) used to refine structure: *SHELXL97* (Sheldrick, 2008 ▶); molecular graphics: *ORTEPII* (Johnson, 1976 ▶) and *DIAMOND* (Brandenburg, 2006 ▶); software used to prepare material for publication: *publCIF* (Westrip, 2009 ▶).

## Supplementary Material

Crystal structure: contains datablocks global, I. DOI: 10.1107/S1600536809049678/hb5241sup1.cif

Structure factors: contains datablocks I. DOI: 10.1107/S1600536809049678/hb5241Isup2.hkl

Additional supplementary materials:  crystallographic information; 3D view; checkCIF report

## Figures and Tables

**Table 1:** Selected bond lengths (Å)

Cd—N4	2.361 (4)
Cd—N3	2.395 (4)
Cd—S1	2.6021 (14)
Cd—S3	2.6310 (15)
Cd—S4	2.7258 (15)
Cd—S2	2.7586 (13)

**Table 2:** Hydrogen-bond geometry (Å, °)

*D*—H⋯*A*	*D*—H	H⋯*A*	*D*⋯*A*	*D*—H⋯*A*
O1—H1o⋯S3^i^	0.84	2.43	3.241 (4)	162
O2—H2o⋯O3^ii^	0.84	1.89	2.723 (5)	176
O3—H3o⋯O4	0.84	1.87	2.688 (5)	166
O4—H4o⋯O2^i^	0.84	1.91	2.745 (5)	174

Symmetry codes: (i) 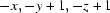; (ii) 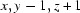.

**Fig. 1.**
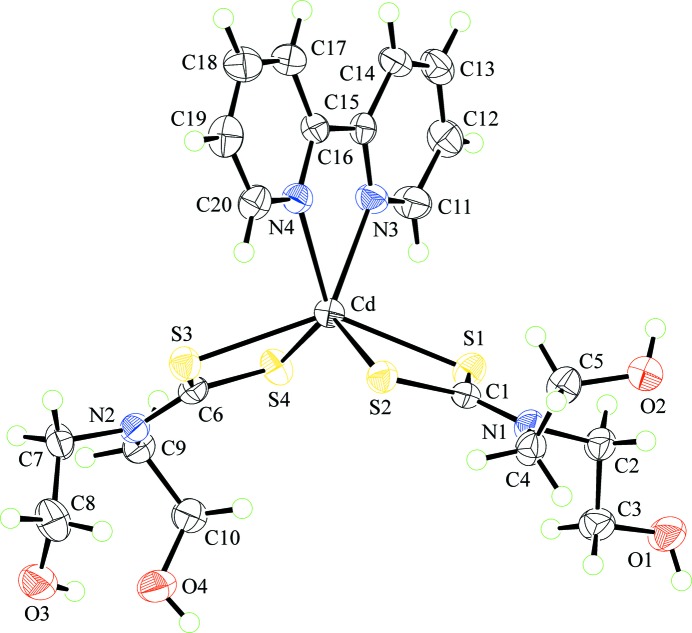
Molecular structure of (I) showing displacement ellipsoids at the 50% probability level.

**Fig. 2.**
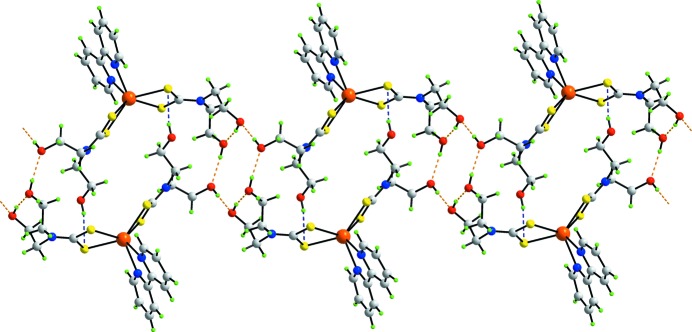
Supramolecular chain in (I) mediated by O–H···O (orange dashed lines) and O–H···S (blue dashed lines) hydrogen bonds. Colour code: Cd, orange; S, yellow; O, red; N, blue; C, grey; and H, green.

## supplementary crystallographic information

### Comment

Interest in the title compound, (I), relates to crystal engineering endeavours with the zinc-triad 1,1-thiolates (Lai *et al.*, 2002; Tiekink, 2003; Chen *et al.*, 2006), in particular with functionalized dithiocarbamate ligands (Benson *et al.*, 2007). The cadmium atom in (I), Fig. 1, is chelated by two dithiocarbamate ligands that form asymmetric Cd–S bond distances (Cd–S1, S2 = 2.6021 (14) and 2.7586 (13) Å; and Cd–S3, S4 = 2.6310 (15) and 2.7258 (15) Å) and by the 2,2'-bipyridyl ligand (Cd–N3, N4 = 2.395 (4) and 2.361 (4) Å). The resulting N_2_S_4_ donor set defines a trigonal prismatic geometry.

The prominent feature of the crystal structure is the formation of a supramolecular chain with base vector [0 1 1]. These form as a result of 12-membered {···OH}_6_ synthons involving the O2-, O3-, and O4-hydroxyl groups; the O1-hydroxyl group forms a hydrogen bond to the S3 atom, Table 1 and Fig. 2. The 12-membered synthons have a flattened chair conformation.

### Experimental

Compound (I) was prepared following the standard literature procedure from the reaction of Cd[S_2_CN(CH_2_CH_2_OH)_2_] and 2,2'-bipyridyl (Lai & Tiekink, 2004). Colourless crystals were obtained from the slow evaporation of a chloroform/ethanol solution of (I).

### Refinement

C-bound H-atoms were placed in calculated positions (C–H 0.95–0.99 Å) and were included in the refinement in the riding model approximation with *U*_iso_(H) set to 1.2*U*_eq_(C). The O-bound H-atoms were located in a difference Fourier map and refined with an O–H restraint of 0.840±0.001 Å, and with *U*_iso_(H) = 1.5*U*_eq_(carrier atom).

### Crystal data

**Table:**

[Cd(C_5_H_10_NO_2_S_2_)_2_(C_10_H_8_N_2_)]	*Z* = 2
*M**_r_* = 629.10	*F*(000) = 640
Triclinic, *P*1	*D*_x_ = 1.645 Mg m^−^^3^
Hall symbol: -P 1	Mo *K*α radiation, λ = 0.71073 Å
*a* = 10.077 (2) Å	Cell parameters from 887 reflections
*b* = 11.568 (2) Å	θ = 4.2–30.2°
*c* = 11.676 (2) Å	µ = 1.22 mm^−^^1^
α = 70.85 (3)°	*T* = 173 K
β = 85.86 (3)°	Plate, colourless
γ = 81.21 (3)°	0.33 × 0.21 × 0.03 mm
*V* = 1270.3 (4) Å^3^	

### Data collection

**Table:**

Rigaku AFC12K/SATURN724 diffractometer	5262 independent reflections
Radiation source: fine-focus sealed tube	4944 reflections with *I* > 2σ(*I*)
graphite	*R*_int_ = 0.073
ω scans	θ_max_ = 26.5°, θ_min_ = 2.7°
Absorption correction: multi-scan (*ABSCOR*; Higashi, 1995)	*h* = −12→11
*T*_min_ = 0.836, *T*_max_ = 1	*k* = −14→14
25287 measured reflections	*l* = −14→14

### Refinement

**Table:**

Refinement on *F*^2^	Primary atom site location: structure-invariant direct methods
Least-squares matrix: full	Secondary atom site location: difference Fourier map
*R*[*F*^2^ > 2σ(*F*^2^)] = 0.053	Hydrogen site location: inferred from neighbouring sites
*wR*(*F*^2^) = 0.116	H-atom parameters constrained
*S* = 1.13	*w* = 1/[σ^2^(*F*_o_^2^) + (0.0386*P*)^2^ + 2.3559*P*] where *P* = (*F*_o_^2^ + 2*F*_c_^2^)/3
5262 reflections	(Δ/σ)_max_ < 0.001
310 parameters	Δρ_max_ = 0.56 e Å^−^^3^
4 restraints	Δρ_min_ = −0.98 e Å^−^^3^

### Special details

**Table:**

Geometry. All e.s.d.'s (except the e.s.d. in the dihedral angle between two l.s. planes) are estimated using the full covariance matrix. The cell e.s.d.'s are taken into account individually in the estimation of e.s.d.'s in distances, angles and torsion angles; correlations between e.s.d.'s in cell parameters are only used when they are defined by crystal symmetry. An approximate (isotropic) treatment of cell e.s.d.'s is used for estimating e.s.d.'s involving l.s. planes.
Refinement. Refinement of *F*^2^ against ALL reflections. The weighted *R*-factor *wR* and goodness of fit *S* are based on *F*^2^, conventional *R*-factors *R* are based on *F*, with *F* set to zero for negative *F*^2^. The threshold expression of *F*^2^ > σ(*F*^2^) is used only for calculating *R*-factors(gt) *etc*. and is not relevant to the choice of reflections for refinement. *R*-factors based on *F*^2^ are statistically about twice as large as those based on *F*, and *R*- factors based on ALL data will be even larger.

### Fractional atomic coordinates and isotropic or equivalent isotropic displacement parameters (Å^2^)

**Table:**

	*x*	*y*	*z*	*U*_iso_*/*U*_eq_	
Cd	0.36192 (3)	0.33563 (3)	0.34083 (3)	0.02981 (11)	
S1	0.14908 (11)	0.27519 (11)	0.47403 (10)	0.0337 (3)	
S2	0.35247 (11)	0.39052 (10)	0.55430 (10)	0.0328 (2)	
S3	0.45303 (11)	0.53668 (10)	0.19791 (10)	0.0337 (3)	
S4	0.23576 (12)	0.43012 (10)	0.12429 (11)	0.0371 (3)	
O1	−0.2255 (3)	0.2988 (3)	0.6889 (4)	0.0461 (9)	
H1O	−0.2940	0.3442	0.7026	0.069*	
O2	0.1836 (3)	0.1573 (3)	0.9494 (3)	0.0422 (8)	
H2O	0.2112	0.0879	0.9418	0.063*	
O3	0.2605 (4)	0.9327 (3)	−0.0806 (4)	0.0497 (9)	
H3O	0.1879	0.9048	−0.0783	0.075*	
O4	0.0530 (3)	0.8094 (3)	−0.0739 (4)	0.0501 (9)	
H4O	−0.0183	0.8255	−0.0369	0.075*	
N1	0.1369 (4)	0.3121 (3)	0.6879 (3)	0.0312 (8)	
N2	0.3187 (3)	0.6426 (3)	−0.0077 (3)	0.0276 (7)	
N3	0.3929 (4)	0.1393 (3)	0.3082 (3)	0.0322 (8)	
N4	0.5818 (3)	0.2321 (3)	0.3877 (3)	0.0290 (8)	
C1	0.2059 (4)	0.3265 (4)	0.5824 (4)	0.0268 (9)	
C2	0.0090 (4)	0.2611 (4)	0.7110 (4)	0.0339 (10)	
H2A	−0.0030	0.2201	0.7993	0.041*	
H2B	0.0120	0.1978	0.6704	0.041*	
C3	−0.1091 (5)	0.3596 (4)	0.6657 (5)	0.0387 (11)	
H3A	−0.1168	0.4212	0.7088	0.046*	
H3B	−0.0980	0.4026	0.5777	0.046*	
C4	0.1797 (5)	0.3524 (4)	0.7844 (4)	0.0340 (10)	
H4A	0.0991	0.3851	0.8236	0.041*	
H4B	0.2337	0.4207	0.7476	0.041*	
C5	0.2614 (5)	0.2511 (4)	0.8802 (4)	0.0385 (11)	
H5A	0.3383	0.2135	0.8407	0.046*	
H5B	0.2976	0.2873	0.9353	0.046*	
C6	0.3334 (4)	0.5458 (4)	0.0943 (4)	0.0291 (9)	
C7	0.4113 (5)	0.7363 (4)	−0.0404 (4)	0.0352 (10)	
H7A	0.4274	0.7629	−0.1293	0.042*	
H7B	0.4985	0.6979	−0.0017	0.042*	
C8	0.3608 (5)	0.8490 (5)	−0.0033 (5)	0.0453 (12)	
H8A	0.3233	0.8219	0.0805	0.054*	
H8B	0.4376	0.8930	−0.0030	0.054*	
C9	0.2229 (4)	0.6488 (4)	−0.1002 (4)	0.0319 (9)	
H9A	0.2328	0.5670	−0.1121	0.038*	
H9B	0.2483	0.7091	−0.1779	0.038*	
C10	0.0770 (5)	0.6847 (4)	−0.0721 (5)	0.0399 (11)	
H10A	0.0204	0.6740	−0.1328	0.048*	
H10B	0.0519	0.6300	0.0088	0.048*	
C11	0.2929 (5)	0.0957 (4)	0.2737 (5)	0.0410 (11)	
H11	0.2104	0.1484	0.2517	0.049*	
C12	0.3044 (5)	−0.0226 (4)	0.2687 (5)	0.0403 (11)	
H12	0.2317	−0.0508	0.2429	0.048*	
C13	0.4236 (5)	−0.0994 (4)	0.3020 (4)	0.0388 (11)	
H13	0.4337	−0.1820	0.3008	0.047*	
C14	0.5278 (5)	−0.0556 (4)	0.3367 (4)	0.0340 (10)	
H14	0.6109	−0.1073	0.3590	0.041*	
C15	0.5105 (4)	0.0651 (4)	0.3389 (4)	0.0282 (9)	
C16	0.6193 (4)	0.1216 (4)	0.3728 (4)	0.0281 (9)	
C17	0.7516 (5)	0.0650 (4)	0.3846 (4)	0.0373 (10)	
H17	0.7762	−0.0133	0.3735	0.045*	
C18	0.8472 (5)	0.1252 (5)	0.4128 (5)	0.0432 (12)	
H18	0.9387	0.0891	0.4204	0.052*	
C19	0.8078 (5)	0.2377 (5)	0.4297 (4)	0.0399 (11)	
H19	0.8717	0.2804	0.4493	0.048*	
C20	0.6736 (4)	0.2881 (4)	0.4177 (4)	0.0341 (10)	
H20	0.6461	0.3649	0.4314	0.041*	

### Atomic displacement parameters (Å^2^)

**Table:**

	*U*^11^	*U*^22^	*U*^33^	*U*^12^	*U*^13^	*U*^23^
Cd	0.03000 (19)	0.02641 (18)	0.0318 (2)	−0.00260 (13)	0.00178 (13)	−0.00888 (13)
S1	0.0301 (5)	0.0409 (6)	0.0335 (6)	−0.0070 (5)	0.0009 (4)	−0.0160 (5)
S2	0.0312 (6)	0.0316 (5)	0.0372 (6)	−0.0072 (4)	0.0034 (5)	−0.0131 (5)
S3	0.0314 (6)	0.0342 (6)	0.0328 (6)	−0.0061 (4)	−0.0029 (5)	−0.0058 (5)
S4	0.0440 (7)	0.0296 (6)	0.0380 (6)	−0.0106 (5)	−0.0019 (5)	−0.0083 (5)
O1	0.0312 (18)	0.043 (2)	0.069 (2)	−0.0080 (15)	0.0054 (17)	−0.0257 (18)
O2	0.0434 (19)	0.0361 (18)	0.045 (2)	−0.0040 (15)	0.0039 (15)	−0.0118 (15)
O3	0.043 (2)	0.0354 (19)	0.069 (3)	−0.0058 (16)	0.0006 (19)	−0.0153 (17)
O4	0.0344 (19)	0.0403 (19)	0.074 (3)	−0.0011 (16)	0.0071 (17)	−0.0205 (18)
N1	0.0284 (18)	0.0336 (19)	0.034 (2)	−0.0080 (15)	0.0035 (15)	−0.0128 (16)
N2	0.0259 (17)	0.0288 (18)	0.0280 (19)	−0.0032 (14)	0.0004 (14)	−0.0092 (14)
N3	0.0322 (19)	0.0258 (18)	0.040 (2)	−0.0037 (15)	−0.0014 (16)	−0.0123 (15)
N4	0.0297 (18)	0.0282 (18)	0.0288 (19)	−0.0033 (14)	−0.0009 (15)	−0.0089 (14)
C1	0.027 (2)	0.0211 (19)	0.031 (2)	−0.0010 (16)	0.0004 (17)	−0.0081 (16)
C2	0.029 (2)	0.034 (2)	0.039 (3)	−0.0073 (18)	0.0023 (19)	−0.0133 (19)
C3	0.035 (2)	0.036 (2)	0.050 (3)	−0.006 (2)	0.002 (2)	−0.021 (2)
C4	0.037 (2)	0.037 (2)	0.034 (2)	−0.0117 (19)	0.0075 (19)	−0.0178 (19)
C5	0.035 (2)	0.047 (3)	0.039 (3)	−0.012 (2)	0.001 (2)	−0.019 (2)
C6	0.028 (2)	0.029 (2)	0.028 (2)	0.0004 (17)	0.0043 (17)	−0.0089 (17)
C7	0.035 (2)	0.031 (2)	0.034 (2)	−0.0097 (19)	−0.0012 (19)	−0.0007 (18)
C8	0.055 (3)	0.038 (3)	0.046 (3)	−0.019 (2)	−0.004 (2)	−0.013 (2)
C9	0.031 (2)	0.035 (2)	0.029 (2)	−0.0030 (18)	−0.0013 (18)	−0.0101 (18)
C10	0.037 (3)	0.036 (2)	0.046 (3)	−0.005 (2)	−0.001 (2)	−0.012 (2)
C11	0.037 (3)	0.034 (2)	0.053 (3)	−0.002 (2)	−0.006 (2)	−0.016 (2)
C12	0.043 (3)	0.037 (3)	0.046 (3)	−0.012 (2)	−0.004 (2)	−0.016 (2)
C13	0.052 (3)	0.029 (2)	0.038 (3)	−0.010 (2)	0.002 (2)	−0.0115 (19)
C14	0.039 (2)	0.023 (2)	0.038 (3)	−0.0010 (18)	−0.002 (2)	−0.0077 (18)
C15	0.031 (2)	0.029 (2)	0.022 (2)	−0.0055 (17)	0.0039 (16)	−0.0052 (16)
C16	0.030 (2)	0.025 (2)	0.026 (2)	−0.0032 (16)	0.0003 (17)	−0.0048 (16)
C17	0.035 (2)	0.033 (2)	0.040 (3)	0.0007 (19)	−0.002 (2)	−0.008 (2)
C18	0.034 (2)	0.043 (3)	0.045 (3)	0.003 (2)	−0.011 (2)	−0.005 (2)
C19	0.036 (2)	0.051 (3)	0.036 (3)	−0.014 (2)	−0.007 (2)	−0.013 (2)
C20	0.035 (2)	0.040 (2)	0.031 (2)	−0.0076 (19)	0.0012 (18)	−0.0156 (19)

### Geometric parameters (Å, °)

**Table:**

Cd—N4	2.361 (4)	C4—C5	1.510 (6)
Cd—N3	2.395 (4)	C4—H4A	0.9900
Cd—S1	2.6021 (14)	C4—H4B	0.9900
Cd—S3	2.6310 (15)	C5—H5A	0.9900
Cd—S4	2.7258 (15)	C5—H5B	0.9900
Cd—S2	2.7586 (13)	C7—C8	1.511 (7)
S1—C1	1.727 (4)	C7—H7A	0.9900
S2—C1	1.715 (4)	C7—H7B	0.9900
S3—C6	1.737 (4)	C8—H8A	0.9900
S4—C6	1.711 (5)	C8—H8B	0.9900
O1—C3	1.423 (6)	C9—C10	1.508 (6)
O1—H1O	0.840	C9—H9A	0.9900
O2—C5	1.428 (6)	C9—H9B	0.9900
O2—H2O	0.839	C10—H10A	0.9900
O3—C8	1.426 (6)	C10—H10B	0.9900
O3—H3O	0.840	C11—C12	1.376 (6)
O4—C10	1.418 (6)	C11—H11	0.9500
O4—H4O	0.839	C12—C13	1.378 (7)
N1—C1	1.343 (5)	C12—H12	0.9500
N1—C4	1.466 (6)	C13—C14	1.372 (6)
N1—C2	1.470 (5)	C13—H13	0.9500
N2—C6	1.339 (5)	C14—C15	1.389 (6)
N2—C9	1.476 (5)	C14—H14	0.9500
N2—C7	1.477 (5)	C15—C16	1.496 (6)
N3—C15	1.349 (5)	C16—C17	1.387 (6)
N3—C11	1.334 (6)	C17—C18	1.386 (7)
N4—C20	1.333 (5)	C17—H17	0.9500
N4—C16	1.341 (5)	C18—C19	1.375 (7)
C2—C3	1.509 (6)	C18—H18	0.9500
C2—H2A	0.9900	C19—C20	1.386 (6)
C2—H2B	0.9900	C19—H19	0.9500
C3—H3A	0.9900	C20—H20	0.9500
C3—H3B	0.9900		
			
N4—Cd—N3	68.54 (12)	C4—C5—H5B	109.2
N4—Cd—S1	124.50 (9)	H5A—C5—H5B	107.9
N3—Cd—S1	89.45 (10)	N2—C6—S4	120.8 (3)
N4—Cd—S3	91.86 (9)	N2—C6—S3	119.9 (3)
N3—Cd—S3	125.71 (10)	S4—C6—S3	119.2 (2)
S1—Cd—S3	138.41 (4)	N2—C7—C8	114.0 (4)
N4—Cd—S4	130.40 (9)	N2—C7—H7A	108.7
N3—Cd—S4	87.33 (10)	C8—C7—H7A	108.7
S1—Cd—S4	96.52 (4)	N2—C7—H7B	108.7
S3—Cd—S4	67.42 (4)	C8—C7—H7B	108.7
N4—Cd—S2	88.63 (9)	H7A—C7—H7B	107.6
N3—Cd—S2	129.89 (10)	O3—C8—C7	113.6 (4)
S1—Cd—S2	67.09 (4)	O3—C8—H8A	108.9
S3—Cd—S2	97.79 (4)	C7—C8—H8A	108.9
S4—Cd—S2	136.68 (4)	O3—C8—H8B	108.9
C1—S1—Cd	89.36 (15)	C7—C8—H8B	108.9
C1—S2—Cd	84.55 (15)	H8A—C8—H8B	107.7
C6—S3—Cd	87.84 (15)	N2—C9—C10	115.8 (4)
C6—S4—Cd	85.31 (15)	N2—C9—H9A	108.3
C3—O1—H1O	111.9	C10—C9—H9A	108.3
C5—O2—H2O	113.2	N2—C9—H9B	108.3
C8—O3—H3O	114.3	C10—C9—H9B	108.3
C10—O4—H4O	113.0	H9A—C9—H9B	107.4
C1—N1—C4	122.5 (4)	O4—C10—C9	110.7 (4)
C1—N1—C2	121.8 (4)	O4—C10—H10A	109.5
C4—N1—C2	115.6 (4)	C9—C10—H10A	109.5
C6—N2—C9	120.8 (4)	O4—C10—H10B	109.5
C6—N2—C7	121.3 (4)	C9—C10—H10B	109.5
C9—N2—C7	117.2 (3)	H10A—C10—H10B	108.1
C15—N3—C11	118.9 (4)	N3—C11—C12	122.7 (4)
C15—N3—Cd	118.6 (3)	N3—C11—H11	118.7
C11—N3—Cd	122.0 (3)	C12—C11—H11	118.7
C20—N4—C16	119.1 (4)	C11—C12—C13	118.5 (4)
C20—N4—Cd	120.3 (3)	C11—C12—H12	120.7
C16—N4—Cd	120.3 (3)	C13—C12—H12	120.7
N1—C1—S2	121.5 (3)	C12—C13—C14	119.5 (4)
N1—C1—S1	119.5 (3)	C12—C13—H13	120.2
S2—C1—S1	118.9 (2)	C14—C13—H13	120.2
N1—C2—C3	112.0 (4)	C15—C14—C13	119.2 (4)
N1—C2—H2A	109.2	C15—C14—H14	120.4
C3—C2—H2A	109.2	C13—C14—H14	120.4
N1—C2—H2B	109.2	N3—C15—C14	121.1 (4)
C3—C2—H2B	109.2	N3—C15—C16	115.9 (4)
H2A—C2—H2B	107.9	C14—C15—C16	123.0 (4)
O1—C3—C2	106.8 (4)	N4—C16—C17	122.0 (4)
O1—C3—H3A	110.4	N4—C16—C15	115.8 (4)
C2—C3—H3A	110.4	C17—C16—C15	122.2 (4)
O1—C3—H3B	110.4	C16—C17—C18	118.6 (4)
C2—C3—H3B	110.4	C16—C17—H17	120.7
H3A—C3—H3B	108.6	C18—C17—H17	120.7
N1—C4—C5	113.7 (4)	C19—C18—C17	119.1 (4)
N1—C4—H4A	108.8	C19—C18—H18	120.4
C5—C4—H4A	108.8	C17—C18—H18	120.4
N1—C4—H4B	108.8	C20—C19—C18	119.1 (4)
C5—C4—H4B	108.8	C20—C19—H19	120.5
H4A—C4—H4B	107.7	C18—C19—H19	120.5
O2—C5—C4	112.1 (4)	N4—C20—C19	122.1 (4)
O2—C5—H5A	109.2	N4—C20—H20	119.0
C4—C5—H5A	109.2	C19—C20—H20	119.0
O2—C5—H5B	109.2		
			
N4—Cd—S1—C1	69.88 (17)	Cd—S1—C1—S2	2.3 (2)
N3—Cd—S1—C1	133.11 (16)	C1—N1—C2—C3	−86.6 (5)
S3—Cd—S1—C1	−76.60 (14)	C4—N1—C2—C3	90.1 (5)
S4—Cd—S1—C1	−139.64 (13)	N1—C2—C3—O1	177.9 (4)
S2—Cd—S1—C1	−1.34 (13)	C1—N1—C4—C5	−95.4 (5)
N4—Cd—S2—C1	−127.34 (16)	C2—N1—C4—C5	87.9 (5)
N3—Cd—S2—C1	−67.13 (18)	N1—C4—C5—O2	−67.0 (5)
S1—Cd—S2—C1	1.36 (13)	C9—N2—C6—S4	−3.3 (5)
S3—Cd—S2—C1	140.97 (14)	C7—N2—C6—S4	−173.7 (3)
S4—Cd—S2—C1	75.79 (14)	C9—N2—C6—S3	176.6 (3)
N4—Cd—S3—C6	136.19 (16)	C7—N2—C6—S3	6.2 (5)
N3—Cd—S3—C6	71.49 (18)	Cd—S4—C6—N2	−175.8 (3)
S1—Cd—S3—C6	−70.90 (15)	Cd—S4—C6—S3	4.3 (2)
S4—Cd—S3—C6	2.65 (14)	Cd—S3—C6—N2	175.6 (3)
S2—Cd—S3—C6	−134.94 (14)	Cd—S3—C6—S4	−4.5 (2)
N4—Cd—S4—C6	−74.77 (18)	C6—N2—C7—C8	−95.9 (5)
N3—Cd—S4—C6	−133.41 (17)	C9—N2—C7—C8	93.4 (5)
S1—Cd—S4—C6	137.46 (14)	N2—C7—C8—O3	−77.0 (5)
S3—Cd—S4—C6	−2.70 (14)	C6—N2—C9—C10	77.5 (5)
S2—Cd—S4—C6	74.19 (15)	C7—N2—C9—C10	−111.8 (4)
N4—Cd—N3—C15	4.5 (3)	N2—C9—C10—O4	67.7 (5)
S1—Cd—N3—C15	−123.2 (3)	C15—N3—C11—C12	0.4 (7)
S3—Cd—N3—C15	80.7 (3)	Cd—N3—C11—C12	−171.9 (4)
S4—Cd—N3—C15	140.2 (3)	N3—C11—C12—C13	0.7 (8)
S2—Cd—N3—C15	−64.3 (3)	C11—C12—C13—C14	−1.2 (7)
N4—Cd—N3—C11	176.8 (4)	C12—C13—C14—C15	0.6 (7)
S1—Cd—N3—C11	49.1 (4)	C11—N3—C15—C14	−0.9 (6)
S3—Cd—N3—C11	−107.0 (4)	Cd—N3—C15—C14	171.6 (3)
S4—Cd—N3—C11	−47.5 (4)	C11—N3—C15—C16	178.1 (4)
S2—Cd—N3—C11	108.1 (4)	Cd—N3—C15—C16	−9.3 (5)
N3—Cd—N4—C20	174.7 (3)	C13—C14—C15—N3	0.4 (7)
S1—Cd—N4—C20	−111.7 (3)	C13—C14—C15—C16	−178.6 (4)
S3—Cd—N4—C20	46.8 (3)	C20—N4—C16—C17	−1.7 (6)
S4—Cd—N4—C20	108.3 (3)	Cd—N4—C16—C17	171.7 (3)
S2—Cd—N4—C20	−51.0 (3)	C20—N4—C16—C15	−179.9 (4)
N3—Cd—N4—C16	1.4 (3)	Cd—N4—C16—C15	−6.4 (5)
S1—Cd—N4—C16	75.0 (3)	N3—C15—C16—N4	10.3 (5)
S3—Cd—N4—C16	−126.6 (3)	C14—C15—C16—N4	−170.7 (4)
S4—Cd—N4—C16	−65.0 (3)	N3—C15—C16—C17	−167.9 (4)
S2—Cd—N4—C16	135.7 (3)	C14—C15—C16—C17	11.2 (6)
C4—N1—C1—S2	1.5 (6)	N4—C16—C17—C18	0.1 (7)
C2—N1—C1—S2	178.0 (3)	C15—C16—C17—C18	178.1 (4)
C4—N1—C1—S1	179.5 (3)	C16—C17—C18—C19	0.8 (7)
C2—N1—C1—S1	−4.1 (5)	C17—C18—C19—C20	−0.1 (7)
Cd—S2—C1—N1	175.8 (3)	C16—N4—C20—C19	2.4 (6)
Cd—S2—C1—S1	−2.2 (2)	Cd—N4—C20—C19	−171.0 (3)
Cd—S1—C1—N1	−175.7 (3)	C18—C19—C20—N4	−1.5 (7)

### Hydrogen-bond geometry (Å, °)

**Table:**

*D*—H···*A*	*D*—H	H···*A*	*D*···*A*	*D*—H···*A*
O1—H1o···S3^i^	0.84	2.43	3.241 (4)	162
O2—H2o···O3^ii^	0.84	1.89	2.723 (5)	176
O3—H3o···O4	0.84	1.87	2.688 (5)	166
O4—H4o···O2^i^	0.84	1.91	2.745 (5)	174

Symmetry codes: (i) −*x*, −*y*+1, −*z*+1; (ii) *x*, *y*−1, *z*+1.
